# Commentaries on the Diseases of Children

**Published:** 1816-03

**Authors:** 


### *Commentaries on the Diseases of Children.*
### By Dr. Clarke.

( Continued from page 168 of our last Number. )

Phrenitis. — This, the most important chapter in the work, shall receive a proportionate attention, and a most extended analysis. Phrenitis is a much more common disease of children than has been suspected; and numbers who are *supposed* to die of idiopathic *convulsions*, are cut off by inflammation of the brain. The following appear to be the principal reasons, why infants are more liable to this complaint than adults; viz. 1st. The disproportioned size of the head in youth, and consequently the greater flow of blood through its vessels. The greater frequency of eruptive disorders about the face and heads of children, proves the greater determination to the head in early life. 2d. The incomplete ossification of the skull, at birth, requires a more abundant supply of blood. 3d. Dentition requires the same. To these may be added, hereditary peculiarities of structure, which may act as predispositions at least, to inflammation of the brain, and its consequence, effusion.

This formidable disease advances under different and insidious modifications. The child sometimes becomes less lively; disposed to quiet rather than amusement; gapes, at an early as well as late period of the day; frowns, and occasionally knits the brows. Starts in sleep, and dreams unpleasantly. These symptoms are too often disregarded by parents, till the accession of violent fever, or a convulsion excites alarm.

In other cases, however, the approach of the malady is more violent, and less equivocal. Great heat, thirst, frequency of pulse (which is generally hard, full, and strong.) White tongue; flushed cheeks, and disturbed sleep, develope themselves. These are often mistaken for symptoms of common fever, and treated as such, till a dilated pupil, strabismus, or convulsion, demonstrates the effusion of water. "Cum mala per longas invaluere moras."

According to the author's experience, fever, independently of a *local cause,* never occurs in the early periods of life. But local disorders, *then,* produce violent effects, and are prone to excite what is termed, inflammatory fever. Peripneumony, phrenitis, indigestible food in the stomach, and many other causes, often excite, suddenly, violent *symptomatic* fever. The *cause* should be investigated, for on a knowledge of *this* the treatment will frequently depend. Although the *general* febrile symptoms are common to phrenitis and many other inflammations, there are some characteristic ones, which may be detected, by moderate attention, in the disease under consideration.

Increased irritability, in the organs of sense, appears very early in phrenitis. The slightest sound will cause the child to start, whether asleep or awake. Sensation becomes more acute; the lightest touch will rouse the little patient with great agitation. Vision becomes painfully sensible; strong light cannot be endured.

"The increased irritability of the retina becomes a diagnostic symptom of inflammation of the brain, at a very early stage of the disease." Page 121.

On the exposure to even a moderate light, the iris will contract violently to such a degree, that a fine needle would not pass the pupil without wounding some of the fibres of the iris. Of the Ancients, Aretæus; of the Moderns, Dr. Clarke, have noticed this symptom.

The tunica conjunctiva of the eye becomes highly vascular. The urinary secretion is diminished, and emits a strong smell. Constipation does not necessarily attend the early stage of infantile phrenitis. Sensible perspiration is interrupted; the skin is intensely hot and arid. These symptoms continue, with little or no interruption, for several days. If dentition with salivation *had* been going on, the saliva now diminishes or ceases.

After a few days, (if convulsions should not occur and give a new character to the disease) the scene changes materially. The increased irritability disappears, and the child becomes weary and indisposed to exertion. Yawning and drowsiness supervene. The sleep, if not interrupted, would continue night and day. Still the heat is above the natural standard; but the exacerbations are now only occasional. The flushings of the cheeks are sometimes very strongly marked, appearing like vermilion. The thirst is inconsiderable, though the mouth remains dry, as do the nostrils. The senses become dull; the hearing less acute; at length the child is found to be insensible to all but loud noises; at last there is total deafness. The sense of feeling follows the same march, and the sleep can scarcely be disturbed. The pulse falls to 110 or 120, seldom lower, becoming less equable, and sometimes interrupted. The pupil now begins to alter, as the retina loses its sensibility; the iris is more expanded; but in the first instance has a vibratory motion. At length, the dilation becomes permanent, the iris losing all power of contraction, even when exposed to the strongest light. The child is then totally blind.

Whilst these changes are going on in the vision, the antagonising muscles of the eye lose their equilibrium, and strabismus is the consequence. This squinting will sometimes continue after recovery. When this state of insensibility has continued some time, a fit of screaming, or convulsions ensue, partial sometimes precede the universal convulsions. In some cases, the fit of screaming or of convulsions comes on in twenty-four or forty-eight hours after the first attack. This fit of screaming (as though the child were cut or torn) lasts, very often, for an hour or more, without intermission;—then the infant remains asleep or quiet for a long time, till a repetition of the attack, which is always followed by farther marks of diminished sensibility. At length the fits of screaming cease, and convulsions, at longer or shorter intervals, continue till death closes the scene; a chronic state of the disease takes place; or remedies remove the cause producing them. After this time, if the cranial ossification was before incomplete, the edges of the separate bones recede from each other; the fontanelles enlarge; and the membranes of ossification will be raised above the general surface.

A convulsion not unfrequently *ushers in* phrenitis, from which the child recovers, and the first stage of the disease developes itself. Screaming, according to Dr. C's experience, "never occurs till after the symptoms of the first stage have been decidedly established." After a fit of this kind, the second stage immediately succeeds. When a patient is cut off in the first stage, which is often the case, it is always by convulsions.

Convulsions, as remarked before, are sometimes universal, sometimes partial; not uncommonly attacking one side, and leaving permanent hemiplegia.

Dr. C. has inspected the heads of patients who had died in every state of progress in this disease. The following are the appearances. When death takes place in the *first* stage, the vessels of the dura and pia mater are found turgid with blood; the plexus choroides highly vascular; the. substance of the brain not decidedly altered; the parts lying on the basis of the skull, generally inflamed: the optic nerves sometimes embedded in a sheath of coagulating lymph; in the ventricles, more than natural quantity of water.

After symptoms of the *second* stage have appeared, and especially where the pupil has been dilated, and where there have been fits of screaming, a considerable effusion will be found between the tunica arachnoides and pia mater or in the ventricles, or in both situations.

The violence of the symptoms is not always commensurate with the derangement of structure found after death. It is often just the reverse. This, Dr. C. thinks, must be owing to difference of irritability in the brain and nervous system of different individuals.

In the second stage of the disease, the peristaltic motion feels the influence of the same torpor that pervades other parts of the body, and obstinate constipation results. From the symptoms above stated, and the *post mortem* appearances, Dr. C. thinks, that the phænomena of the different stages admit of explanation. Thus, in the most early stages, when there is a trifling addition to the quantity and momentum of blood circulating through the brain, the irritability of that organ and the nerves connected with it becomes increased; hence the increased excitement of the touch, vision, and hearing. The increased energy of the brain produces a corresponding state in the heart and arteries; hence the frequent, hard, and strong pulse, &c. The external and internal carotid arteries being branches of the same trunk, any increased impetus to the head will affect both sets of vessels; hence, when the interior of the skull is very much loaded with blood, the scalp frequently becomes very red from the communication of vessels by anastomosis. This circumstance accounts for convulsions during dentition, when it proceeds with strong inflammatory symptoms. Since blood coming from the heart by a common trunk to the gums and brain, pressure is made on the latter from fulness of the vessels, *without extravasation of blood or water.* The *latter* is proved by single paroxysms occurring, from which the child recovers the use of all its faculties in an hour or two, which could not be the case had blood or water been *extravasated.**

* Dr. C. has seen women in puerperal convulsions first attacked with pain in the head; this has become more and more violent till it ended in

"The screaming fits probably take place with the first effusion of water between the membranes, or in the ventricles of the brain. Between the unyielding skull and the incompressible fluid, the substance of the brain and the origin of all the nerves connected with it are violently compressed, as if in a vice, till the veins carry off some of the blood, by which the brain is set at liberty, in some measure, and the pain is lessened. A farther effusion produces a repetition of similar symptoms, till at length the brain becomes insensensible to farther pressure from the increasing effusion, and the patient lies in a torpid state till he dies." 138.

Although this explanation may not be deemed quite satisfactory, we have seen none better, or indeed so good.

Dr. C. thinks, that the predisposition to this disorder is to be found partly in original structure, "which may consist, perhaps, in a greater capaciousness of the blood-vessels, together with a laxity of their coats; a circumstance likely to happen in scrofulous subjects. It is certain, that the children of scrofulous parents, *cæteris paribus*, are most liable to the disease." 139. If his experience may be trusted, the disease has also its predisposition in hereditary structure. It is of great importance in practice therefore, to enquire whether any child of the family had previously died of convulsions attended with fever.

Another source of *predisposition* is the unnaturally warm clothing kept about the heads of children, by which too much blood is invited to the brain, even before the process of dentition goes on rapidly; this last itself is a predisposition to phrenitis.

The *occasional* causes, (independently of local violence) are the same as in other inflammations of internal organs; especially sudden atmospherical vicissitudes. The *modus operandi* of dentition; indigestible food in the stomach, &c. acting as occasional causes of inflammation in the brain, however explained, need not be doubted in point of fact. There may, however, be other hidden predispositions, in which mere fulness of the vessels, with strong action of the heart are sufficient to produce the effect.

Inflammation of the brain, in all cases, and in all stages, is so formidable and fatal a disease, that many practiti-

---

total blindness; then convulsions, with loss of powers of the mind, have succeeded. Blood has been plentifully taken away; the head has been raised and kept cold, and the patient purged copiously and delivered. The convulsions have immediately ceased; the sight has returned, and the mind has become strong in a few hours. This could not have happened had extravasation taken place.

oners maintain, that it seldom, if ever, admits of cure in the first, and never in the second stage. Dr. Clarke however contends, " that *often* in the first stage, if early treated, and *sometimes* even in the second stage, the disease admits of remedy." The prognostic, therefore, should not be too gloomy, lest exertion should be checked, and the child left to die.

" When symptoms of the second stage (hydrocephalus) have taken place without screaming, the danger appears to be less, and the chance of relief by the use of remedies, greater."

The treatment of this inflammation has been less successful than that of most other internal inflammations, partly because the practitioner is not often called in till a dangerous progress is made in the disease, and partly because the diagnostic symptoms are frequently overlooked by superficial observers. The disease is mistaken for common fever, or that which precedes eruptive disorders, (especially the measles, as impatience of light is common to both) for common head-ache, for intestinal fever, and for dentition. Here procrastination takes away all chance of success. On the other hand, if the diagnostic symptoms be attended to, if the history of the disease be accurately investigated, " this disease, in its first stage, will, if early treated, yield to the remedies employed, *like other internal inflammations;* and so its consequence (hydrocephalus internus) may be prevented." 147.

" Inflammations of the brain from external violence frequently give way, as surgeons well know ; and why not, when arising from other causes ?"

From this we dissent, because the *predisposition* is not so likely to be present in phrenitis from accident, as in spontaneous inflammation of the sensorium.

Of all known remedies for phlegmonous inflammation, copious bleedings from a large orifice, suddenly performed, are the most powerful. The same practice is applicable to inflammation of the brain. But as, in children, a large vessel can seldom be found, the scarificators or leeches must be substituted. To the operation of cupping, Dr. C. gives a decided preference, and deplores the want of dexterity in performing this operation, among general practitioners, and deprecates the repugnance evinced towards it by parents and friends.

" There should be no compromise in cases of health and life; and he ill consults the value of his own professional character, but above all, the approval of his own mind, who commutes the safety of the patient, (to acquire a short-lived popularity) for the character of compliance with the wishes and fears of parents, however natural! When the present danger has ceased, and the cool exercise of their understandings returns, they will have very different feelings, and will have lost all their confidence in the skill of one who submitted to be controuled by those whom he should himself have directed."

And deservedly will he lose their confidence, say we!

On the promptness and efficacy of blood-letting, in these cases, every thing depends. Very young children bear well the loss of blood, even to fainting, once or twice, but they ill bear a more frequent repetition.

"From a child of seven or eight months, two ounces and a half of blood may be taken; and one and one half, or two more, in sixteen hours afterwards. Three ounces may be taken from a child of a year old, and two and a half, or three afterwards, if the symptoms do not yield; at this age two more may be taken in twelve hours after the second bleeding, if necessary, and the child has not been too much weakened already. Cupping may be performed on the scalp, behind the ears, nape of the neck, high up on the spine, or between the shoulders." 153.

At and after one year, the external jugulars will often be sufficiently large and superficial to admit venæsection. As the child becomes older, the brachial veins become prominent. Dr. C. gives a decided preference to venæsection from the external jugular. The anatomical reasons are obvious, and Dr. C. has needlessly enlarged upon them. From peculiarities of circulation through the brain, the blood is easily detained there; and when local plethora has once been induced, it is longer before it admits of remedy, and requires greater perseverance both of patient and practitioner, than a similar accumulation of blood in any other part, although here assisted by the operation of gravitation. The peculiarities alluded to, are the structure of the sinuses, and the manner in which the blood from the internal and external jugulars enters the cava superior.

In opening the jugular vein, "it is best to make an incision in the first instance, through the skin, longer than the intended orifice in the vein, so as to expose the vein distinctly; and then to make an incision into the vein itself: by this means a thrombus will be avoided. Without these precautions, the vein is very apt to roll, the incision does not enter it, or a small orifice only is made." If neither the jugular nor brachial veins can be opened, then recourse must be had to cupping. The rectum should be immediately opened by a glyster of salts and senna; while the stomach and intestines should be forthwith emptied, by giving a large dose of calomel, and in two hours afterwards a purgative of neutral salts in infusion of senna, with manna. The dose should be repeated every two or three hours, until plentiful evacuations have been procured.

" The use of saline purgatives is particularly insitsed upon, because they occasion a large watery secretion from the bowels, and in this manner co-operate with the operation of bleeding, in reducing the quantity of the circulating blood, and so diminishing the pressure on the head directly, besides their indirect effect in causing a revulsion from the head to a distant part of the body."

Two or three watery evacuations from the intestines should every day afterwards be procured. Between the exhibitions of purgatives, ipecacuanha or antimony should be exhibited every four hours, in such doses, as to diffuse the circulation over the surface of the body; and, if possible, induce a state of perspiration.

The erect posture should be maintained, for the sake of gravitation. The heads of very young children should be kept much raised during the day, and at night they should be propped up on pillows stuffed with horse hair, or chaff, to avoid the warmth of the feathers. Blisters to the scalp Dr. C. justly condemns, as inviting blood to the head, when it is already in excess there. Blisters to the outsides of the legs he found eminently beneficial, especially when the denuded surfaces are kept open by the cerat. sabinæ. Blisters, however, may be safely applied to the upper part of the back; while cataplasms, so as to act as rubefacients, may be put to the lower extremities. The latter, in all cases of inflamed brain, should be kept very warm. To the head, *cold* is recommended to be applied by our author, who relates cases where it was of decisive efficacy.

" The principle on which the application of cold was proposed, and the manner of its agency may, perhaps, be thus explained. The head being kept very high and cold, less blood will be carried to it by the carotid arteries; at any rate, less will circulate through the external carotid arteries; the external jugular veins will necessarily contain a smaller quantity, and their stream will not oppose, in the same degree, that which enters the superior vena cava by the internal jugular veins. The sinuses of the dura mater will, by this means, enjoy a more free discharge of the blood contained in them, and that by the veins of the pia mater, a more ready admission into the sinuses.

" Whether the principle be right or wrong, the writer is certain of the practical advantage of the application of cold, having used it in multiplied instances since that time, both in cases of fullness of the vessels of the brain, in cases of puerperal convulsions, in inflammation of the brain, and in epilepsy. When it has been employed in children of an age to be sensible of the effects of it, or in adults, they have always desired to have it often repeated, from finding the advantage and comfort of it. The child who was seen by Dr. Baillie, was perpetually calling for it to mitigate her pain. When ice or snow cannot be conveniently procured, a mixture o. spirit, æther, and water, immersed in a cold medium, is a very good substitute, and cloths wetted with it may be applied constantly. The advantage arising from it does not rest upon his own experience only, but on that of various medical practitioners, who have, on his recommendation, of late years, had recourse to it. It is applicable not only to this case of inflammation of the brain, but to all cases in which it is desirable to diminish the flow of blood to the brain." 173.

Tepid bathing, by diffusing the circulation, is a useful auxiliary; but it requires great care that the surface of the body be not exposed to cold upon coming out of the water; with this precaution, it may be used twice a-day, and an hour at a time.

As to food, it is needless to remark, that nothing of the kind should be exhibited during the inflammatory stage; even pure liquids are to be restricted, lest by augmenting the mass of fluids in circulation, they increase the pressure on the brain.

The effusion of water into the ventricles, or between the membranes, in the second stage of the disease, appears to be the natural cure of the first, or inflammatory state, and is common to this and the inflammation of many other circumscribed cavities, as the pericardium, thorax, abdomen, &c. In some, as the ovarium and tunica vaginalis, the effusion does not interfere with any function of life, and becomes inconvenient only from its bulk. Even in ascites, the functions of the viscera will often proceed tolerably, notwithstanding the presence of water. In hydrothorax and hydropericardium, the effused fluids, of course, interfere with the most important actions, and are proportionally more dangerous and distressing. The bony structure of the skull, like that of the thorax, increases the bad effects of the pressure, and in a still greater degree, because it presses on all sides equally. Incomplete ossification, for obvious reasons, renders the symptoms of hydrocephalus more mild at first, and more slow in developing themselves, than where the head is completely ossified.

When the symptoms above described as indicative of effusion, particularly squinting, dilated pupil, screaming, &c. have occurred, the measures pursued for the cure of the first stage must be discontinued. The absorption of the fluid effused, and the prevention of a farther effusion are now the indications. The *first* of these is very difficultly effected. Diuretics are uncertain; neutral salts, squill, elaterium, have seldom produced any decided advantage.

"Mercury appears to be the only medicine which has been successful in the cure of acute hydrocephalus; and with this view it may be used both externally and internally, in very considerable quantities." Dr. C. justly observes, "that from the doses being estimated as bearing some proportion to those which may be employed in adults, it has been less useful than it might have been."

Without pretending that mercury has any *specific* operation on hydrocephalus, our author remarks, that,

"It is not too much to observe, that its effects in relieving this disorder are not to be explained by any principle, or any analogy to its effects in other cases of accumulation of water in circumscribed cavities. It is not in proof that mercury rubbed on the thighs and back, has proved a cure for dropsy of the pericardium, thorax, abdomen, tunica vaginalis, or even a collection of redundant fluid in a joint; yet, it is on record, and the writer has had various opportunities of witnessing its pre-eminently good effects in hydrocephalus, succeeding inflammation of the brain." 180.

The dread of salivation has deterred many practitioners from employing mercury in large quantities with children. Dr. C. asserts, however, that although "he has prescribed mercury in very large quantities, in a great number of cases, he never produced salivation, except in three instances, in any child under three years of age. But, admitting that a profuse salivation should occur, there is no comparison between that and the danger of the disease." 182.

As every moment is precious in the treatment of this complaint, it is necessary to use measures the most prompt and decisive. At any age above one year, half a drachm of the ung. hyd. fort. with five grains of camphor, may be rubbed in, every six hours, on any broad surface of the body. In forty-eight hours, this may be increased to two scruples or a drachm. The back is the best surface, from its breadth; next to that, the thighs, on their inside, from the number of absorbents. Every day the dried ointment should be washed off, and the parts rubbed again when dried. In respect to the internal administration of mercury, the author does not speak decidedly, as he never uses it independently of frictions.

"To children of a year old and upwards, one grain of calomel may be given every six, four, or even three hours, unless diarrhœa should supervene. It most commonly brings on a discharge of green mucous stools. When this happens, the internal use of mercury may be discontinued, or the dose lessened." 183.

The evidence of favourable operation from mercury will be the amelioration of some of the symptoms of the second stage, as the squinting, dilated pupil, deafness, blindness, &c. The flattening of the tumor at the anterior fontanelle is also a favourable symptom. Dr. C. has seen the fontanelle change from convex to concave in the progress to recovery. No precise time can be laid down for the continuance of the mercury; but it must be kept in view, that the disease never admits of a natural cure, and must prove fatal unless remedies make an impression on it. A patient under Mr. Jennet recovered after more than a year, during the whole of which time, with little intermission, mercury had been used by friction. In the course of the treatment, she was quite an idiot; but at the end of a twelvemonth she recovered her understanding. Epilepsy succeeded in this case.

However unpromising the second stage (or actual effusion) may be, every thing should be attempted that offers the smallest chance of success. Inaction and despair combined, never yet did any good in human affairs. Unexpected changes sometimes occur in diseases, and it is difficult (not to say impossible) to circumscribe the powers of the constitution, when assisted by the judicious application of art.

*Idiotism, Paralysis, and Epilepsy, in Children.*

Idiotism not unfrequently occurs as the sequel of inflammation of the brain in children, and becomes chronic. It probably results from some unknown alterations in the configurations or relative position of the brain, not sufficient to destroy life, but enough to weaken the faculties of the mind, or rather to annihilate them. "For this state the writer knows no remedy." 188.

Paralysis, of some part or parts of the body, is another consequence of cerebral derangement from pressure or inflammation. It is always a distressing, generally an unpromising, but sometimes a medicable disorder. Perpetual blisters to the back are occasionally useful: so are issues or setons applied near to the head; friction of the paralysed parts, frequently employed, and long continued; stimulating embrocations; the use of electricity.

When the child is old enough to be sensible of the disadvantage of a paralytic limb, attempts at voluntary motion should be made, and long persevered in. If these measures be neglected, the child becomes a cripple during life.

*Epilepsy, or Chronic Convulsions,* sometimes also remain after the cessation of inflammation of the brain in children, and the absorption of the water which had been effused during the continuance of it. Epilepsy arising from these causes is sometimes, though rarely, removed entirely. But the intervals of the paroxysms admit of being considerably increased, by paying a very strict attention to diet, especially by enjoining abstinence from animal food, a too nourishing regimen, as well as every description of fermented liquor; by sleeping with the head very much elevated; by early rising; by keeping the hair short, the head cold, and the rest of the body very warm; by avoiding all exposure of the body to cold, and maintaining a regular and free course of the bowels.

Setons and issues may also be employed with advantage in cases of epilepsy as well as in paralysis. The daily use of a tepid bath, from 86 to 94, will, by diffusing the circulation, likewise be serviceable. At first, the time of immersion may be ten minutes, which may be afterwards extended to an hour.

Epilepsy sometimes happens to children from great irritation in the alimentary canal, by accumulation of fæculent matter in the stomach and bowels, and from the round and tape-worm.

When epilepsy occurs without symptoms of direct pressure on the brain, and where there are sickness, flatulence, disturbed sleep, and marks of disordered digestion, either preceding or following the paroxysms, it will be right to evacuate the contents of the stomach by an emetic, consisting of sulphate of zinc in an aqueous infusion of ipecacuan, and to repeat it in six, eight, or ten days, according to circumstances.*

To a child of four years, six, eight, or ten grains of the sulphate of zinc, in half an ounce of an infusion of fifteen grains of ipecacuan in an ounce of boiling water. The dose, how-

---

* Dr. C. lately saw a case of epilepsy in a boy, which yielded to the treatment here recommended. Being of a full habit, blood was taken, by cupping, from the scalp, he was purged briskly, and confined to slender diet. Every week, after the symptoms of plethora were subdued, he took an emetic of zinci sulphas, in an infusion of ipecacuan. Under this plan, the frequency of the fits was reduced, and he has now remained many months free from a paroxysm, though before the adoption of it they returned every week, and sometimes oftener.

ever, must vary, according to the age and different degrees of irritability of the stomach Afterwards, in debility of the stomach, light tonic bitters, with carbonate of ammonia, or ol. cajuputi, may be exhibited. Where acidities prevail, soda, or liquor potassæ, may be combined with the bitter. When costiveness is present, magnesia may be employed; if the contrary state is the case, the cretaceous powders. Preparations of iron are dangerous in children, as promoting too great a circulation in the head, and too great an action of the heart.

Preparations of zinc are not liable to the same objection; but they should not be given in doses that will be apt to excite nausea and vomiting. In a child of two years old, the dose of sulphate of zinc will range from one to three grains; of white oxyd of zinc, from one to six grains.

"When the paroxysms of chronic convulsion are preceded by head-ache, sleepiness, and general torpor, accompanied by sighing or frequent yawning, particularly in robust subjects, blood should be immediately taken away, by opening the jugular vein; or by cupping from the scalp behind the ears, or from the nape of the neck. Purgatives of quick action should be often exhibited, and the diet should be reduced, and the head should be kept cold and elevated.

"In some instances epilepsy exists without any apparent affection of the head or stomach.

"These are, however, very rare; and, by attention, the connexion between it and some derangement of the functions of these parts, may often be discovered, though it might not at first have been detected."

"The writer has known two instances in which the sulphate of zinc was of great service in relieving chronic epilepsy of many years standing in adults. One of these was in a gentleman engaged in the profession of the law, a patient of Dr. Reynolds and Mr. Peter Howith, of Chancery Lane. He had generally some slight warning of the approach of the paroxysms; and he was ordered, instantly on perceiving them, to take half a drachm of sulphate of zinc in an infusion of ipecacuanha, as an emetic of quick action. By continuing this plan for some time, he escaped the fits altogether.

"The success of this case led to trying it on a man who was a private tutor, and with whose employment the frequent occurrence of epileptic fits greatly interfered. He had very little notice of the approach of the fits; but as they occurred frequently, that is, about once in three weeks, he was directed to take a similar emetic once in a week; afterwards, once in a fortnight; then every three weeks; and ultimately once in a month. For two years he altogether escaped the paroxysms, and considered himself well. He now became impatient of the inconvenience of taking the emetics, and, notwithstanding the solicitation of his friends, he discontinued them altogether. For some time he continued free from the disease, but at length was suddenly attacked by a paroxysm when walking a small distance from London, and died.—The result of these cases certainly leads to the probability of the advantage which may, in some instances, arise from using this remedy.

"Immersion of the whole body in cold water has been tried as a remedy for cases of epilepsy. But experience does not appear to justify a recourse to it; and if the principles which have been laid down in these Commentaries are founded in truth, it must be injurious, and cannot in any way be useful. It will necessarily have the effect of repelling the blood from the surface to the interior of the body, and the vessels of the head must be rendered more full. Under these circumstances, if there should be any organic defect of the brain, or of the exterior of the skull, upon which the epileptic paroxysm depends, an immediate attack may be expected, or at any rate a foundation will be laid for future paroxysms."

To the preceding analysis we have allotted a space proportioned to the utility rather than the size of the work. We have strictly fulfilled the intentions of the Medico-Chirurgical Reviewers, in presenting their readers with a luminous and condensed portrait of the work reviewed; serving alike as a stimulus and inducement to place the *original* in the library, where it is convenient; and where not so, serving as a useful succedaneum and correct reference. We shall anxiously look for the completion of these Commentaries by the relatives of their worthy but departed author.

---

